# Introducing Human *APOE* into A**β** Transgenic Mouse Models

**DOI:** 10.4061/2011/810981

**Published:** 2011-10-19

**Authors:** Leon M. Tai, Katherine L. Youmans, Lisa Jungbauer, Chunjiang Yu, Mary Jo LaDu

**Affiliations:** ^1^Department of Anatomy and Cell Biology, University of Illinois at Chicago, Chicago, IL 60612, USA; ^2^Translational Neurobiology, Medtronic Incorporated, Minneapolis, MN 55432, USA

## Abstract

Apolipoprotein E (apoE) and apoE/amyloid-*β* (A*β*) transgenic (Tg) mouse models are critical to understanding apoE-isoform effects on Alzheimer's disease risk. Compared to wild type, apoE^−/−^ mice exhibit neuronal deficits, similar to apoE4-Tg compared to apoE3-Tg mice, providing a model for A*β*-independent apoE effects on neurodegeneration. To determine the effects of apoE on A*β*-induced neuropathology, apoE^−/−^ mice were crossed with A*β*-Tg mice, resulting in a significant delay in plaque deposition. Surprisingly, crossing human-apoE-Tg mice with apoE^−/−^/A*β*-Tg mice further delayed plaque deposition, which eventually developed in apoE4/A*β*-Tg mice prior to apoE3/A*β*-Tg. One approach to address h*APOE*-induced temporal delay in A*β* pathology is an additional insult, like head injury. Another is crossing human-apoE-Tg mice with A*β*-Tg mice that have rapid-onset A*β* pathology. For example, because 5xFAD mice develop plaques by 2 months, the prediction is that human-apoE/5xFAD-Tg mice develop plaques around 6 months and 12 months before other human-apoE/A*β*-Tg mice. Thus, tractable models for human-apoE/A*β*-Tg mice continue to evolve.

## 1. Introduction

Alzheimer's disease (AD) is the most common form of dementia and represents a serious economic and social burden worldwide. The familial form of AD (FAD) is caused by autosomal dominant mutations that increase levels of the 42 amino acid isoform of the amyloid-beta 42 (A*β*42) peptide [1, 2]. The primary genetic risk factor for AD is inheritance of the *APOE4* gene for apolipoprotein E (apoE), compared to *APOE3*, with *APOE2* reducing risk [3–5]. The mechanism(s) by which apoE and A*β* affect pathogenesis of the disease is unclear (reviewed [3–5]). However, evidence suggests that apoE may isoform-specifically modulate A*β*-induced neurotoxicity [4, 5]. To address potential mechanisms *in vivo*, several transgenic (Tg) mouse models have been developed to assess the structural and functional interactions between apoE and A*β*. However, each of these models has potential drawbacks that affect the interpretation and physiological relevance of the results. This paper will summarize the current models resulting from the introduction of human-*APOE* into A*β*-Tg mice. The continued development and characterization of both apoE and apoE/A*β*-Tg mouse models is critical to understanding the apoE-isoform effects on AD pathology.

### 1.1. Amyloid-*β*

Traditional diagnosis of AD is based on pathology that includes extracellular amyloid plaques, composed primarily of A*β*42, intraneuronal neurofibrillary tangles, hyperphosphorylated tau, neuroinflammation, and neuronal cell loss. A*β* is a 39–43 amino acid (4 kDa) peptide produced via sequential proteolysis of amyloid precursor protein (APP) by *β*-secretase/BACE followed by *γ*-secretase (composed of presenilins [PS] 1 and 2), to produce A*β* peptides primarily 40 and 42 amino acids in length. Genetic and experimental evidence indicates that A*β*42 is the cause of AD pathogenesis [1, 2]: (1) FAD, although only 3–5% of all AD cases is caused by autosomal dominant mutations in APP, PS1, or PS2 that increase levels of A*β*42 or the A*β*42 : 40 ratio; (2) Down syndrome is caused by trisomy of chromosome 21 (the location of the APP gene), and is characterized by plaque deposition and dementia by the age 40; (3) A*β*42 is neurotoxic *in vitro* and *in vivo*. A*β*, particularly the more toxic A*β*42, aggregates to form a variety of higher-order assemblies including oligomers, protofibrils, fibrils, and amyloid plaques [6]. Amyloid plaques themselves exist in different conformations including compact plaques (composed of a dense Thioflavin-S- (ThioS-) positive core), neuritic plaques (identified as ThioS-positive plaques surrounded by a ring of dystrophic neurites), and diffuse plaques (characterized by amorphous wisps of amyloid that lack a central core and are not neurotoxic) [7–10]. While total plaque burden does not directly correlate with dementia [11], it is an indication of increased A*β*42 levels, and compact or dense core plaques may be disease relevant [7, 12].

### 1.2. ApoE

ApoE is the only apolipoprotein synthesized within the blood-brain barrier (BBB) and is the primary apolipoprotein associated with lipoprotein particles in the central nervous system (CNS), as peripheral apoE is unable to cross the BBB or blood-cerebrospinal fluid barrier. While the majority of apoE in the CNS is secreted by glial cells, particularly astrocytes, neuronal production of apoE has been observed under specific pathological conditions [13]. CNS lipoproteins are critical for lipid homeostasis, particularly as cholesterol and phospholipids are required for neuronal growth, repair, and synaptogenesis (reviewed [3–5]). In humans, three apoE polymorphic alleles exist (*APOE2*, *APOE3,* and *APOE4)* which encode three protein isoforms (apoE2, apoE3, and apoE4). Although human-apoE (h-apoE) is a 299 amino acid protein, the three apoE-isoforms differ by a single amino acid substitution at residues 112 or 158: apoE2 contains Cys^112,158^, apoE3 contains Cys^112^Arg^118^, and apoE4 contains Arg^112,158^ [5]. In the general population, *APOE3* is the most common allele (77%), followed by *APOE4 *(15%) and *APOE2* (8%). In contrast, 40% of AD patients express at least one *APOE4* allele. Compared to *APOE3*, inheritance of one or two copies of the *APOE4* allele increases the risk for developing AD by 4- and 12-fold, whereas *APOE2* decreases risk by 2- and 4-fold [5, 14]. Amyloid plaque deposition is greatest in AD patients with an *APOE4* allele(s) [14]. In addition to AD, *APOE4* is a risk factor for cerebral amyloid angiopathy (CAA; amyloid deposition in blood vessels) and impairs recovery from cerebral insults such as stroke, cerebral hemorrhage, and brain injury [4]. While apoE knock-out mice (apoE^−/−^) and h-apoE-Tg models demonstrate that apoE affects neuronal viability independent of A*β*-induced neurotoxicity, the focus of this paper is on the synergism between the h-apoE isoforms and A*β* on neuropathology. To address the latter *in vivo*, apoE/A*β*-Tg models were subsequently developed to specifically address the isoform-specific effects of h-apoE on A*β* deposition.

## 2. Transgenic Mice Expressing Human ApoE (Table 1)

Several approaches have been used to make Tg mouse models to assess the function of apoE. ApoE^−/−^ Tg-mice were initially used to help understand the role of apoE in the brain [15, 16] although the homology between mouse (m-) and h-apoE is 70%, and mice express only a single isoform, comparable to apoE4 at residues 112 and 158 [54]. ApoE^−/−^ mice have been used as the background for several h-*APOE*-Tg mouse lines. Heterologous promoters have been used to drive the expression of h-apoE in glia or neurons. Examples include glial fibrillar acidic protein (GFAP; glial) [22, 55–57], transferrin (neuronal) [58], platelet-derived growth factor (PDGF; neuronal) [59], neuron-specific enolase (NSE; neuronal) [15], and thymocyte differentiation antigen 1 (Thy1; neuronal) [59]. However, these models have limitations inherent in the use of a heterologous promoter and specific to apoE: (1) the expression of protein by a heterologous promoter is not regulated as it would be by the endogenous promoter; (2) the inserted copy number of the transgene cannot be regulated by a traditional Tg mouse approach; (3) while the cell-specific expression of apoE in the brain is controversial [60–63], the majority of evidence suggests that glia, not neurons, are the primary cell type to express apoE [13, 64–67]; (4) by using the m-apoE^−/−^ background and inducing apoE expression via CNS-specific promoters, lack of peripheral apoE becomes a variable of potential importance when interpreting results from these mice; (5) evidence suggests that in both humans and apoE-TR mice, apoE4 levels are significantly lower than apoE2 or apoE3 [68–73]. Knock-in or targeted-replacement (TR) mice were developed that express h-apoE under the control of the endogenous mouse promoter and provide an alternative to heterologous expression of h-apoE. In apoE-TR mice, the coding domain for each of the h-*APOE* isoforms replaces the coding domain for m-*APOE*. Thus, in apoE-TR mice, glial cells express h-apoE in a native conformation at physiologically regulated levels, and in the same temporal and spatial pattern as endogenous m-apoE. Thus, the interpretation of apoE isoform-specific results is determined by the nature of the apoE-Tg mouse model.

As discussed, a number of apoE-Tg mouse models have been developed with apoE expression under the control of different promoters [74]. The general phenotypes of apoE^−/−^ mice and three examples of the most widely studied apoE-Tg mice (GFAP-apoE, NSE-apoE, and apoE-TR) are briefly discussed (Table 1). 

### 2.1. Apo*E* ^−/−^

Compared to wild type, apoE^−/−^ mice have decreased excitatory transmission, spine density, and dendritic length [16–18]. These changes may underlie behavioral deficits, as apoE^−/−^ mice are cognitively impaired [15, 19, 20]. However, lack of peripheral apoE can have profound effects on plasma lipid homeostasis, potentially leading to a number of confounding variables, including metabolic disease and increased risk for cardiovascular disease, compromising the ability to compensate for oxidative stress or inflammation, deficits that can effect the vasculature of the brain. In addition, the relevance of these mice is unclear as there are no apoE^−/−^ humans. ApoE^−/−^ provides the background for a number of the h-apoE-Tg mice.

### 2.2. GFAP-apoE Mice

GFAP is an intermediate filament protein, expressed at high levels by glia in the CNS. To target apoE expression to glia, mice expressing h-apoE under the control of the GFAP promoter were crossed with apoE^−/−^ mice [17, 22, 23]. At the cellular level, GFAP-apoE4 mice show increased CA1 cellular atrophy and decreased spine density compared to GFAP-apoE3 mice [75]. In addition, compared to GFAP-apoE3 mice, GFAP-apoE4 mice demonstrate impaired cognition and increased anxiety; common symptoms in AD patients [17, 23, 24]. Interestingly, cognitive impairment is evident at an earlier age in female compared to male GFAP-apoE4 mice [23, 24]. A limitation of this model is that the effect of apoE isoform on neuroinflammatory responses cannot be interpreted, as apoE is expressed under the control of a promoter that is induced by neuroinflammation.

### 2.3. Neuronal apoE Expression

Neuronal apoE expression remains controversial, although expression has been identified under conditions of stress [25]. To investigate the role of neuronal expression, apoE-Tg mice expressing h-apoE under the control of the NSE promoter were crossed with apoE^−/−^ mice [25–27]. Kainic acid was used to induce apoE expression in these mice and resulted in the protection of NSE-apoE3 mice from age- and kainic acid-induced presynaptic and dendritic degeneration compared to NSE-apoE4 mice [25]. In addition, female NSE-apoE4 mice demonstrate impaired cognition compared to female NSE-apoE3 mice [25]. In contrast, male NSE-apoE4 mice do not exhibit cognitive deficits [15]. Thus, the effect of apoE4 on cognition may be modulated by gender. This is similar to the early development of cognitive deficits in GFAP-apoE4.

### 2.4. ApoE-TR

To investigate the function(s) of apoE in a more physiologically relevant apoE-Tg model, apoE-TR mice were developed to express each h-apoE isoform under the control of the endogenous m-*APOE* promoter [28–35]. Although apoE protein levels in brain homogenates of these mice were initially described as being similar for each h-apoE isoform [32], subsequent analysis demonstrated that apoE4 levels are significantly lower in plasma, CSF, and brain homogenates than apoE2 and apoE3 [28, 30], similar to the levels of apoE isoforms in humans [68–73]. Compared to apoE3-TR mice, apoE4-TR mice demonstrate decreased spine density and dendrite length, reduced excitatory transmission, and long-term potentiation (Table 1). Again, similar to the cognitive deficits observed in female GFAP-apoE4 mice and NSE-apoE4 mice, female apoE4-TR mice are cognitively impaired compared to female apoE3-TR mice [31, 32]. However, male apoE3-TR and apoE4-TR mice are not significantly different. Interestingly, both apoE3- and apoE4-TR females are cognitively impaired when compared to apoE-isoform-matched males.

## 3. ApoE in A*β* Transgenic Models (Table 2)

### 3.1. A*β*-Tg

Although amyloid plaques are a hallmark of AD, the mechanisms underlying A*β*-induced toxicity remain unknown. To help determine the effects of A*β* and amyloid deposition on neurotoxicity *in vivo*, A*β*-Tg mouse models have been produced that express human FAD mutations. These models include but are not limited to: PDAPP (APP^V717F^), Tg2576 (APP^K670N, M671L^), J9 (PS1^M146V,L286V^, APP^V717F^), as well as 5xFAD (APP^K670N, M671L,I716V,V717I^, PS1^M146V,L286V^) (reviewed in [2]). These A*β*-Tg mice express either APP mutations alone (PDAPP, Tg2576) or in combination with PS mutations (J9, 5xFAD). Plaque development generally begins at 6 months of age in these mice (with the exception of 5xFAD), and the onset of cognitive deficits is dependent on the model (e.g., PDAPP at 6 months, Tg2576 at 9 months). Because the APP^K670N, M671L^ mutation is linked primarily to CAA, the Tg2576 mice also develop A*β* deposition around blood vessels, particularly leptomeningeal and cortical vessels. However, one limitation of A*β*-Tg models is the relative lack of neuronal loss as observed in AD (reviewed [76]) although there are some exceptions (e.g., 5xFAD [49], APP^SL^PS1KI, and TBA2 mice [76]).

### 3.2. Apo*E* ^−/−^/A*β*-Tg

Initially, the effect of apoE on A*β* deposition was investigated using apoE^−/−^ crossed with A*β*-Tg mice (Table 2). Specifically, apoE^−/−^ mice were crossed with PDAPP [77–79] or Tg2576 mice [80]. In both models, the absence of apoE significantly delayed ThioS-positive plaque deposition by 6 months and decreased A*β* levels in the hippocampus and cortex, as measured biochemically from brain homogenates and by A*β* immunoreactivity [42, 77] (Table 2). *APOE* affects plaque deposition in a gene-dose-dependent manner, as plaque levels were intermediate in apoE^+/−^/PDAPP compared with apoE^+/+^/PDAPP and apoE^−/−^/PDAPP mice [78]. In addition, the A*β*40 CAA present in the Tg2576 mice was absent in the apoE^−/−^/Tg2576 mice [80].

### 3.3. GFAP-apoE/A*β*-Tg

The initial studies demonstrating that the lack of m-*APOE* delayed plaque deposition in A*β*-Tg mice led to the question of what would be the effect of introducing h-*APOE* into apoE^−/−^/A*β*-Tg mice (Table 2). To initially address this issue, GFAP-apoE mice was crossed with apoE^−/−^/PDAPP mice [41, 42]. Surprisingly, the introduction of h-*APOE* did not result in the expected reduction in the age of onset of A*β* pathology, rather the presence of h-*APOE* further delayed A*β* deposition. Amyloid accumulation is delayed from 6 to 12 months in apoE^+/−^/PDAPP mice, and to 15 months in GFAP-apoE^+/−^/PDAPP mice (Table 2). Once plaque pathology returned, the greatest plaque burden was found in GFAP-apoE4/PDAPP mice, compared with GFAP-apoE2/PDAPP and GFAP-apoE3/PDAPP mice. One potential confounding factor in these mice, as well as the NSE-apoE mice described below, is the equal expression of the h-*APOE* isoforms, in contrast to human data where inheritance of an *APOE4* allele results in lower apoE levels [68, 69, 73].

### 3.4. Neuronal apoE Expression/A*β*-Tg

To determine the effect of neuronal h-*APOE* expression on A*β* pathology, NSE-apoE were crossed with J9 mice (PS1^M146V,L286V^, APP^V717F^) [81]. Similar to GFAP-apoE/PDAPP-Tg mice, the introduction of h-*APOE* to J9 mice delayed plaque pathology from 8 to 19 months, with deposition greater in the NSE-apoE4/J9 than NSE-apoE3/J9 [26].

### 3.5. ApoE-TR/A*β*-Tg

The physiologic advantages of using apoE-TR mice to study the function(s) of apoE *in vivo* led to the generation of apoE-TR/PDAPP [30] and apoE-TR/Tg2576 mice [47] (Table 2). The resulting data confirmed that h-*APOE* delayed plaque deposition. In apoE-TR/PDAPP mice, plaque deposition was delayed from 6 months to 18 months of age. An apoE isoform-specific effect on A*β* pathology was also observed, with ThioS staining, A*β* immunoreactivity, and A*β* levels in brain homogenates highest with apoE4 [30]. In Tg2576 mice, plaque deposition initiates at 9 months of age, while in apoE-TR/Tg2576 mice there is minimal plaque staining at 15 months. Interestingly, at 15 months, the isoform effect in these mice is primarily on CAA (E4 > E3), as amyloid deposition in the parenchyma is minimal [47].

### 3.6. Addressing h-APOE-Induced Delay in A*β* Pathology

The major drawback to the apoE/A*β*-Tg crosses described thus far is the significant h-*APOE*-induced delay in A*β* pathology. For example, in apoE-TR/PDAPP mice, plaque deposition is not identified until mice are ≥18 months of age (Table 2). This substantial delay precludes timely analyses of apoE isoform-specific effects on early aspects of A*β* pathology. One approach to address this temporal delay is to introduce an additional insult, such as traumatic brain injury (TBI) [82], kainic acid [25], nitric-oxide-synthase-2- (NOS2-) knock-out [83], or by blocking A*β* degradation via neprilysin inhibition [84]. Although no amyloid deposition is present in 12-month-old GFAP-apoE/PDAPP mice, TBI at 9 months leads to amyloid deposition at 12 months [82], which is greater with apoE4 compared to apoE3. Kainic acid decreases synaptophysin and MAP-2 staining in apoE^−/−^ and NSE-apoE Tg mice, with the effect more pronounced with apoE4 than apoE3. Nitric oxide, produced by inducible NOS (encoded for by the NOS2 gene), is an important signaling and redox factor that plays a key role in neuroinflammation and neurodegeneration [83]. NOS2^−/−^ mice have been crossed with multiple A*β*-Tg mouse models, and results demonstrate increased tau phosphorylation and neuronal loss in NOS2^−/−^/A*β*-Tg mice [83]. In the Tg2576/NOS2^−/−^ mice, A*β* deposition is higher compared to Tg2576 mice. Inhibition of neprilysin, an enzyme that degrades extracellular A*β*, with thiorphan, induces fibrillization and deposition of A*β* and in wild-type mice. Thiorphan treatment of apoE-TR mice leads to aggregation of mouse A*β* 1 week after treatment, with higher A*β* deposition in apoE4-TR compared to apoE3-TR mice [84, 85]. Thus, thiorphan treatment of h-apoE-A*β*-Tg mice represents a potential method of accelerating human A*β* deposition. 

An alternative method to address the h-*APOE*-induced temporal delay in A*β* accumulation is to cross apoE-TR mice with A*β*-Tg mice that have rapid-onset A*β* pathology, such as 5xFAD [49], 3xTR [86, 87], APP^SL^PS1KI [88], APPPS1 [89], or TgCRND [90]. For example, because 5xFAD mice develop plaques by 2 months, the prediction is that apoE-TR/5xFAD-Tg mice develop plaques around 6 months and 12 months before other human-apoE/A*β*-Tg mice (LaDu lab, unpublished observations and [50–53]). The approaches described herein will lead to more tractable apoE/A*β*-Tg-models to assess the apoE isoform-specific effects on A*β* pathology.

## 4. Concluding Remarks

ApoE is the greatest risk factor for AD. ApoE^−/−^ mice exhibit neuronal and cognitive deficits. Human apoE-Tg mouse models demonstrate that, compared to apoE3, apoE4 increases markers of neurodegeneration and cognitive impairment. Initially, to determine the effect of apoE on A*β* pathology, A*β*-Tg mice were crossed with apoE^−/−^ mice, resulting in a significant delay in plaque deposition. Surprisingly, the introduction of h-*APOE* to several apoE^−/−^/A*β*-Tg mouse models further delayed plaque deposition. This temporal delay restricts the usefulness of the current apoE/A*β*-Tg mice for investigating the process of A*β* accumulation and the resulting neurotoxicity. To accelerate A*β* deposition, current apoE/A*β*-Tg models could be treated with an additional insult such as TBI, crossed with other Tg models of neurodegeneration (NOS2^−/−^), or treated with drugs that decrease A*β* degradation. Alternatively, the development of A*β* pathology could be accelerated by crossing A*β*-Tg models with a rapid onset of A*β* pathology, such as 5xFAD mice with apoE-TR mice. Transgenic models such as these provide tractable models for identifying biomarkers and the efficient initial validation of therapeutic targets.

## Figures and Tables

**Table 1:** *ApoE* transgenic models. Summary of the effects induced by deletion of mouse apoE (apoE^−/−^) or the introduction of h-apoE isoforms on markers of neuronal degeneration and behavior. *Key*: HC: hippocampus; CX: cortex; DG: dentate gyrus; ChAT: choline acetyl transferase; ICH: intracerebral hemorrhage; LTP: long-term potentiation; TR: targeted replacement (knock-in); F: female; M: male; m: month.

ApoE model	[ApoE]	Neurodegeneration	Behavioral phenotype	Gender effects on behavior	References
ApoE^−/−^	NA	*Spine density and dendritic length*: WT > KO (HC/DG, 12 m)	*Water maze*: WT > KO (6 m, F and M)	*Water maze*: M > F (6 m)	[15–21]
		*ChAT*: WT > KO (HC, 6 m, M)	*Anxiety vertical exploratory*: KO > WT (6 m, F)		

GFAP-apoE	Matched	*Spine length and density*: E3 > E4 (HC, 12 m)	*Water maze*: E3 > E4 (6 m, F)	*Water maze*: M > F (apoE4 only)	[17, 22–24]
			*Radial arm maze*: E3 > E4 (11–14 m, M)		

NSE-apoE	Matched	*Synaptophysin*: E3 > E4 (HC, 5 m)	*Water maze*: E3 > E4 (6 m, F)	*Water maze*: M > F (apoE4 only, 6 m)	
		*ChAT activity*: E3 > E4 = KO (12 m):			[25–27]
		*Tau phosphorylation*: E4 > E3 (9 m)			
		Kainic acid induced neurodegeneration *Synaptophysin and MAP-2*: E3 > E4 = KO (CX, 5 m)			[25]

ApoE-TR	E2 ≥ E3 > E4	*Spine density and length*: E3 > E4 (1, 3 and 12 m)	*Water maze/Active Y maze avoidance*: E3 > E4 (15 m, F)	*Water maze*: M > F (6 and 13 m)	
		*Dendritic length and node number*: E3 > E4 (1 m)	*Anxiety elevated platform*: E4 > E3 (6–8 m, F)		
		*EPSP interval*: E4/E4 > E2/E4 > E3/E3 (1 m)			[18, 28–35]
		*LTP*: E3 > E4 (2–4 m)			
		*ICH*: E4 > E3 (15 m+)			
		*Vascular amyloid*: E4 > E3 (15 m+)			

**Table 2:** *APOE *in A*β* transgenic models. Genetic deletion of mouse apoE (apoE^−/−^) from A*β*-Tg mice delays amyloid deposition, which is further delayed by the introduction of h-*APOE* into A*β*-Tg mice. *LaDu Lab unpublished observations.

		A*β* pathology (age of onset)				
Transgenic line	2–4 months	4–8 months	8–10 months	11–13 months	≥13 months	Total A*β* levels
PDAPP^+/−^ [36–39]		*Diffuse Aβ* * staining*: hippocampus cortex	*ThioS (moderate)*: hippocampus cortex	*ThioS (heavy)*: hippocampus cortex	*ThioS (heavier)*: hippocampus cortex	
PDAPP^+/−^ × apoE^+/−^ [40]				*ThioS (low)*: hippocampus cortex	*ThioS (moderate)*: hippocampus cortex	
PDAPP^+/−^ × apoE^−/−^[41]				*Diffuse Aβ* * staining: * hippocampus	*Thios (low): * hippocampus cortex	*6, 12, 15, 18, and 21 months:* PDAPP^+/−^ > PDAPP^+/−^/apoE^−/−^
PDAPP^+/−^ × GFAP-apoE^+/−^ [42]					*Thios (moderate): * hippocampus E4 > E3 > E2	*6, 12, 15, 18, and 21 months: * E4 > E3
PDAPP^+/+^ × apoE-TR [43]					*ThioS low: * hippocampus E4 > E3 = E2 cortex E4 > E3 > E2	*3 and 12 months: * E4 > E3

Tg2576 [42, 44–46]			*Thios (low): * hippocampus cortex	*ThioS (moderate): * hippocampus cortex	*ThioS (heavy): * hippocampus cortex	
			*CAA-fibrillar (low): * leptomeningeal and cortical vessels	*CAA-fibrillar (heavy): * leptomeningeal and cortical vessels	*CAA-fibrillar (heavy): * leptomeningeal and cortical vessels	
Tg2576 × apoE^−/−^ [42]				*Diffuse Aβ* * staining*: hippocampus cortex		*12 months *Tg2576 > Tg2576/apoE^−/−^
Tg2576 × apoE-TR[47]					*CAA-fibrillar (moderate): * leptomeningeal vessels E4 > E3	

J9 mice [48]			*ThioS (moderate): * cortex hippocampus			
J9 X NSE-apoE [26]					*ThioS: * hippocampus E4 > E3	

5xFAD [49]	*ThioS (moderate): * hippocampus cortex	*ThioS (heavy): * hippocampus cortex				
*5xFAD × apoE-TR [50–53]		*ThioS (low): * hippocampus cortex				
